# Metformin: A Dual-Role Player in Cancer Treatment and Prevention: A Comprehensive Systematic Review and Meta-Analysis

**DOI:** 10.3390/medicina61061021

**Published:** 2025-05-30

**Authors:** Imran Rangraze, Adil Farooq Wali, Mohamed El-Tanani, Mohamed Anas Patni, Syed Arman Rabbani, Rasha Babiker, Shakta Mani Satyam, Yahia El-Tanani, Manfredi Rizzo

**Affiliations:** 1 Internal Medicine Department, Ras Al Khaimah College of Medical Sciences, Ras Al Khaimah Medical and Health Sciences University, Ras Al Khaimah P.O. Box 11172, United Arab Emirates; 2 College of Pharmacy, Ras Al Khaimah Medical and Health Sciences University, Ras Al Khaimah P.O. Box 11172, United Arab Emirates; 3 Community Medicine, College of Medicine, Ras Al Khaimah Medical and Health Sciences University, Ras Al Khaimah P.O. Box 11172, United Arab Emirates; 4 Physiology, College of Medicine, Ras Al Khaimah Medical and Health Sciences University, Ras Al Khaimah P.O. Box 11172, United Arab Emirates; 5 Pharmacology, College of Medicine, Ras Al Khaimah Medical and Health Sciences University, Ras Al Khaimah P.O. Box 11172, United Arab Emirates; 6 Royal Cornwall Hospital Trust, NHS, Truro TR1 3LJ, UK; 7 Department of Endocrinology, School of Medicine, University of Palermo, 90133 Palermo, Italy

**Keywords:** metformin, cancer, treatment, prevention

## Abstract

*Background and Objectives*: Metformin is said to reduce the incidences and deaths resulting from cancer in patients suffering from type 2 diabetes mellitus, but the results have been inconsistent. Perform a systematic review and meta-analysis concentrating on the different outcomes of several cancers while taking into account the impact of metformin use. *Materials and Methods*: As of 15 October 2024, the literature for Medline, Embase, and Web of Science was systematically searched. ROBINS-I and the RoB 2 tool were used for assessing the risk of bias in observational studies and randomized controlled trials (RCTs), respectively. The strength of the evidence with respect to the GRADE criteria was checked. Random effects meta-analyses were conducted alongside sensitivity analyses, subgroup analyses, and meta-regressions. By utilizing funnel plots as well as Egger’s test and trim-and-fill analysis, publication bias was evaluated. *Results*: In total, 65 studies were included in the final analyses: Metformin intake was linked to a lower risk of cancer (RR 0.72; 95% CI: 0.64–0.81, I^2^ = 45%). Significant reductions were observed in breast cancer (RR 0.68; 95% CI: 0.55–0.83) and colorectal cancers (RR 0.62; 95% CI: 0.51–0.76). Evidence certainty fluctuated from moderate to low, though analyses confirmed the results. Plofs funded the publication bias, but adjustment in trim-and-fill did not change the outcome significantly. *Conclusions*: Metformin intake seems to lower the chances of developing several types of cancers, especially breast and colorectal cancers, but the observational designs hinder determining the causal factors for observational studies. There is a need for large RCTs.

## 1. Introduction

Cancer remains a leading cause of morbidity and mortality worldwide, with an urgent need for effective preventive and therapeutic strategies. Emerging evidence suggests that metabolic modulators, such as metformin, may offer potential benefits beyond glycemic control. Metformin, a biguanide class antihyperglycemic agent widely prescribed for type 2 diabetes mellitus (T2DM), exerts its primary effect by activating AMP-activated protein kinase (AMPK) pathways, leading to reduced hepatic glucose production and enhanced insulin sensitivity [1,2,3,4].

In preclinical models, metformin demonstrated anticancer properties, including inhibition of cell proliferation, induction of apoptosis, and modulation of inflammatory and metabolic signaling pathways [5,6]. Observational studies and randomized controlled trials explored the association between metformin use and various cancer outcomes, yet the findings remain inconsistent, partly due to heterogeneity in study designs, populations, cancer types, and methodological limitations such as confounding by indication [7].

Given these inconsistencies and the growing clinical interest, a comprehensive and methodologically rigorous systematic review and meta-analysis is warranted. This study aims to synthesize the available evidence on the relationship between metformin use and cancer incidence, mortality, and survival outcomes, while rigorously evaluating the quality of evidence, addressing sources of heterogeneity, and providing a clear interpretation of the strength and limitations of the existing literature.

## 2. Materials and Methods

### 2.1. Search Strategy

From the earliest possible date until October 2024, thorough literature search was conducted. Databases searched: Medline (via PubMed), Embase, and Web of Science.

Date of final search: 15 October 2024.

Search string used (Medline):

(“Metformin”[Mesh] OR “Metformin”[tiab]) AND (“Neoplasms”[Mesh] OR “Cancer”[tiab] OR “Carcinoma”[tiab]) AND (“Incidence”[Mesh] OR “Mortality”[Mesh] OR “Survival”[Mesh] OR “Prevention”[Mesh]).

No language or time restrictions were applied.

The subsequent MeSH phrases and/or keywords either used alone or combination of these were utilized:
CancerMetforminNeoplasmsPreventionCarcinomaRiskIncidenceBisguanidesType two DiabetesHypoglycaemic agentsChemotherapyMetformin monotherapyMortality

Only human epidemiological research was included in the search. Furthermore, current review references were examined for pertinent articles. If there were several publications covering an identical topic, the most recently published and comprehensive one was selected.

Compliance with Reporting Guidelines:

This systematic review and meta-analysis was conducted in accordance with the Preferred Reporting Items for Systematic Reviews and Meta-Analyses (PRISMA) 2020 guidelines.

A completed PRISMA 2020 checklist and flow diagram have been included to ensure transparent and comprehensive reporting.

### 2.2. Inclusion Criteria

- RCTs and observational studies.
- Patients exposed to metformin compared to placebo, other therapies, or no therapy.
- Outcomes: cancer incidence, mortality, or treatment response.
- Reported effect estimates (RR, OR, and HR) with 95% CI.

### 2.3. Exclusion Criteria

- Animal studies.
- Reviews, commentaries, and incomplete abstracts.
- Studies lacking sufficient data for effect estimates.

### 2.4. Study Selection and Data Extraction

Two independent reviewers screened and extracted data. Discrepancies were resolved by consensus or a third reviewer.

The following information was taken out of every study: authors with year, study design, sample size, country, comparison groups, outcome, and conclusion. The relative risk or odds ratio (95 percent confidence interval) was employed as standard association metric.

The Cochrane Risk-of-Bias Analyzer 1.0 was employed for examining the scientific content of every reported RCT. The level of research of the investigation was also measured using the Jadad rating. Every study’s potential for bias was evaluated by the two researchers using five criteria: selective reporting, insufficient outcome data, blinding, allocation concealment, and random sequence creation.

Risk of Bias Assessment

- RCTs: evaluated using the RoB 2 tool.
- Observational studies: evaluated using the ROBINS-I tool.
- Summary presented in tables (ROBINS-I Table 1, RoB 2 Table 2).
- Certainty of evidence: the GRADE methodology was used to assess the certainty of evidence for each major cancer outcome.

### 2.5. Data Synthesis and Statistical Analysis

Random-effects models were used to pool results. Heterogeneity was assessed with I^2^ and τ^2^ statistics. Sensitivity analyses excluding high-risk studies were performed. Subgroup analyses were conducted by cancer type, geographic location, and study design. Meta-regression was conducted where ≥10 studies were available.

Assessment of publication bias: publication bias was evaluated via funnel plots, Egger’s regression test, and the trim-and-fill method.

## 3. Results

### 3.1. Results of Literature Search

#### 3.1.1. Study Identification Through Databases and Registrations

There were 82 records found in the databases. There were 20 records found in the registers. Twelve duplicate records were eliminated. Eight records were flagged as inappropriate by automation tools. Eleven records were deleted for additional reasons. Seventy-one records were examined. Twelve records were excluded. A total of 59 reports were requested to be retrieved. Five records’ reports were not retrieved. In total, 54 reports were evaluated for eligibility. Reports (n = 8) were removed due to improper handling of study results (n = 3), lack of risk ratio data (n = 2), and just one research investigation for a particular kind of cancer (n = 3). Forty-six records were ultimately chosen.

#### 3.1.2. Identification of Studies via Other Methods

**Figure 1:**
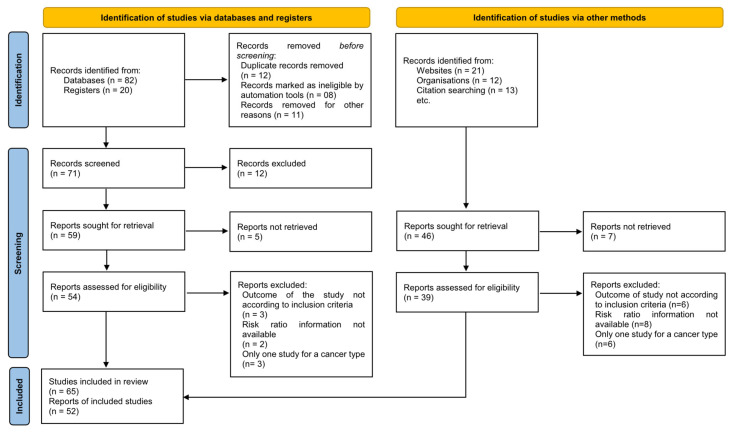
PRISMA flow chart demonstrating selection of studies in this systematic review and meta-analysis.

In total, 21, 12, and 13 (n = 46) records were found through citation searches, websites, and organizations. Forty-six reports were requested to be retrieved. Seven studies’ reports were not retrieved. Thirty-nine reports were evaluated for eligibility. Reports (n = 20) were removed due to improper handling of study results (n = 6), lack of risk ratio data (n = 8), and just one research investigation for specific kinds of cancer (n = 6). Nineteen records were ultimately chosen. There were 65 studies in the review. There were 52 reports of the included studies (Figure 1 and Table 1).

#### 3.1.3. Overview of Included Studies

The included studies were published between 2006 and 2024. Studies included in this systematic review had different comparison groups, such as metformin users being compared with non-metformin users [18,23,24], metformin users being compared to other anti-hyperglycemic users [27,37], metformin monotherapy for cancer treatment being compared against placebo [11,21], and metformin + other chemotherapeutic drugs for cancer treatment compared to placebo [7,8,9]. The outcomes evaluated were cancer prevention, risk of cancer development, and cancer treatment. Some studies focused on all types of cancers [6,56,57], while some studies focused specifically on “prostate cancer” [29,37], “breast cancer” [17,23,30], “pancreatic cancer” [10,13], “colorectal cancer” [24,42], and other cancers [7,9,31].

### 3.2. Qualitative Analysis

#### 3.2.1. Role of Metformin in Cancer Prevention, Risk of Cancer Development, and Cancer Treatment in All Types of Cancer

O’Connor L and associates [5], in a 2024 meta-analysis, concluded that metformin may lower the incidence of several cancers, but the results are not as reliable due to substantial variability and the possibility of bias in publications [RR 0.56; 95% CI (0.31–0.81)]. Orchard and associates, in 2023 [6], observed that metformin consumption had been linked to lower risk of cancer among older persons with diabetes who lived in the community. More investigation is required for assessing whether metformin consumers who received random access to aspirin showed higher possibility of cancer death.

Zhang and associates, in 2021 [56], conducted a systematic review as well as a meta-analysis that showed that metformin could serve as a stand-alone preventive factor against cancer risk in people with T2D. Kim and associates, in 2018 [25], conducted research involving metformin users as well as non-metformin users. According to results of this research, persons with T2D who take metformin exhibited lower risk of acquiring cancer [RR 0.524; 95% CI (0.319–0.827)]. Franciosi and associates, in 2013 [58], performed research evaluating metformin’s role in overall mortality in different cancers. The findings imply that metformin may be linked to a notable decrease in probability of cancer and deaths related to cancer [RR 0.68; 95% CI (0.54–0.81)].

Geraldine and associates, in 2012 [44], concluded that patients suffering from diabetes are more susceptible to develop malignancy in relation to those without the disease. Furthermore, metformin and other antidiabetic medications were linked to decreased likelihood of cancer in males with diabetes. Monami and associates, in 2011 [46], observed that another compelling argument for continuing metformin therapy among individuals receiving insulin treatment may be a reduced likelihood of malignant tumors.

Bowker and associates, in 2010 [51], carried out research to evaluate the role of metformin in overall mortality in different types of cancers. They found that metformin treatment is more linked to a lower likelihood of cancer events than sulfonylurea treatment [RR 0.81; 95% CI (0.66–0.99)]. According to Libby and associates in 2009 [52], using metformin may lower the likelihood of developing cancer [RR 0.64; 95% CI (0.54–0.76)]. Currie and associates, in 2009 [54], analyzed the impact of monotherapy with metformin or sulfonylurea, combined therapy (metformin + sulfonylurea), or insulin in any type of cancer. Malignancies were more common in people taking insulin or secretagogues of insulin than in people on metformin; the majority of this increased risk was eliminated when metformin was used in conjunction with insulin [RR 0.56; 95% CI (0.44–0.67)]. According to Bowker and associates in 2006 [55], patients experiencing type 2 diabetes who were subjected to exogenous insulin and sulfonylureas were at considerably higher risk of dying from cancer than those who were administered metformin [RR 0.82; 95% CI (0.61–0.91)].

Ramos-Penafiel C and associates, in 2018 [21], carried out research on metformin chemotherapy for cancer treatment. All types of cancer treatment were evaluated. They found good results with metformin aligned with chemotherapy.

#### 3.2.2. Metformin Role in Cancer Risk, Treatment, and Prevention in Breast Cancer

Hoiso and associates, in 2019 [17], performed research on metformin users, other forms of ADM, as well as statin users regarding breast cancer prevention. There was no proof that the frequency of breast cancer in females suffering from T2D was correlated with the administration of statins or metformin. There was a marginally higher frequency of breast cancer among insulin users [RR 0.98; 95% CI (0.90–1.06)]. Tang and associates, in 2018 [23], in their meta-analysis, found that individuals with T2D experiencing breast cancer may have a higher cumulative survival rate if they take metformin. There was no discernible instance of metformin affecting the frequency of breast cancer [RR 0.975; 95% CI (0.772–1.232)].

Calip and associates, in 2016 [30], evaluated metformin users, sulfonylureas users, and insulin users in a group of breast cancer patients. Research findings are inconsistent with the formerly postulated reduced likelihood of breast cancer associated with taking metformin, or the higher risk associated with insulin utilization [RR 0.96; 95% CI (0.52–1.78)]. Bosco and associates [45], in their 2011 study, concluded that for women having T2D who are perimenopausal or postmenopausal, metformin may offer safeguards against breast cancer [RR 0.82; 95% CI (0.64–0.97)].

Bodmer and associates, in 2010 [49], found that long-term application of metformin by women having T2D was linked to lower incidence of cancer of the breast [RR 0.45; 95% CI (0.23–0.83)]. Serageldin M.A. and associates, in 2023 [8], conducted a clinical trial where metformin + AC-T was evaluated in breast cancer treatment as compared to a placebo. They observed better control of chemotherapy-induced toxicities [RR 1.23; 95% CI (0.90–1.46)].

Goodwin P.J. and associates [11], in their 2023 study of metformin monotherapy for breast cancer treatment, found that the DFS was not improved by metformin in vulnerable surgical manageable BC [RR 0.99; 95% CI (0.90–1.00)]. Bakry H.M. and associates, in 2023 [12], evaluated metformin + paclitaxel in breast cancer treatment. Remarkable protection against paclitaxel-induced PN [RR 1.45; 95% CI (1.23–1.65)] was observed.

**Figure 2:**
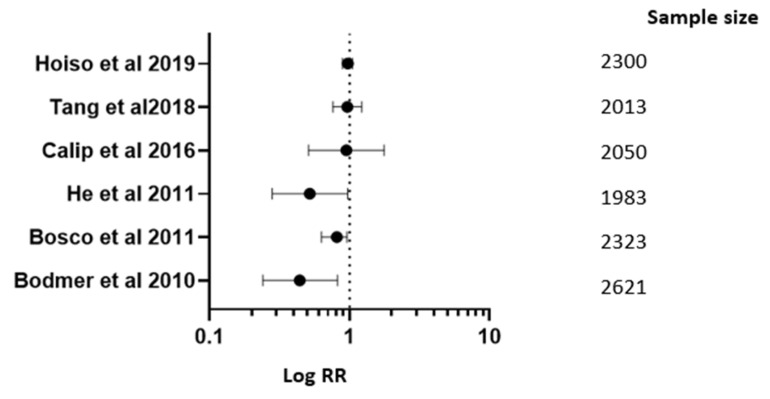
Forest plot graph for metformin’s role in cancer risk, treatment, and prevention in breast cancer [17,23,30,42,45,49].

Lord S.R. and associates, in 2018 [22], evaluated metformin monotherapy for breast cancer treatment. There was remarkable reduction in overall incidence. Sonnenblick A. and associates, in 2017 [28], evaluated metformin + trastuzumab, lapatinib, or their combination for breast cancer treatment. Research shows that the danger of endometrial cancer was not significantly impacted by metformin usage, insulin usage, or other antidiabetic medications (Table 1 and Table 2, Figure 2).

#### 3.2.3. Metformin Role in Cancer Risk, Treatment, and Prevention in Prostate Cancer

Kuo and associates, in 2019 [18], studied metformin users as well as non-metformin users in prostate cancer prevention. It was concluded that for men with diabetes and BPH, taking metformin may lower their risk of developing prostate cancer [RR 0.70; 95% CI (0.50–0.97)]. Häggström and associates, in 2016 [29], evaluated metformin users, sulfonylurea users, and insulin users in prostate cancer prevention. The findings provide no evidence in favor of the recently proposed theory that metformin prevents prostate cancer. The findings did, however, provide some evidence for a negative association with the risk of prostate cancer as well as the level of severity of T2DM [RR 0.97; 95% CI (0.78–1.20)].

According to Preston and associates in 2014 [37], although people with diabetes taking alternative oral hypoglycemics did not have a lower chance of PCa development, usage of metformin was linked to lower risk [RR 0.86; 95% CI (0.76–0.98)]. According to Wright and associates in 2009 [53], in Caucasians, taking metformin had been linked to substantial decrease in general likelihood of PCa.

**Figure 3:**
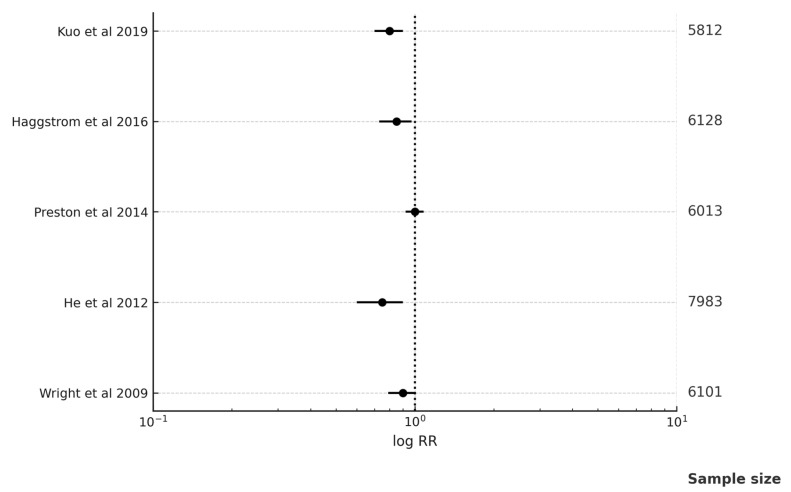
Forest plot for metformin role in cancer risk, treatment, and prevention in prostate cancer [18,29,37,41,53].

Galsky M.D. and associates, in 2017 [26], conducted a clinical trial to evaluate metformin monotherapy for prostate cancer treatment. They concluded that metformin was well-tolerated and demonstrated minimal anti-PCa activity [RR 1.46; 95% CI (1.26–1.66)]. Pujalte Martin M et al. (2021) [15] carried out a clinical trial involving metformin + docetaxel in prostate cancer treatment. The combined chemotherapy failed to improve the outcome [RR 0.99; 95% CI (0.90–1.10)] [Table 1 and Table 3, Figure 3].

#### 3.2.4. Metformin Role in Cancer Risk, Treatment, and Prevention in Pancreatic Cancer

Hu and associates, in 2023 [10], carried out a case control study comparing metformin non-users and metformin users in pancreatic cancer prevention. It was concluded that metformin consumers who suffer from diabetes can lower their chances of pancreatic cancer.

Kim and associates, in 2022 [13], evaluated DM patients using metformin, DM patients not using metformin, and non-DM patients at risk of pancreatic cancer development. It was observed that compared to women with diabetes who do not take metformin, women with diabetes who take metformin are more likely to develop pancreatic cancer. On the other hand, men with diabetes mellitus who employ metformin are just as likely to develop cancer of the pancreas as those who do not [RR 1.127; 95% CI (0.660–1.934)].

**Figure 4:**
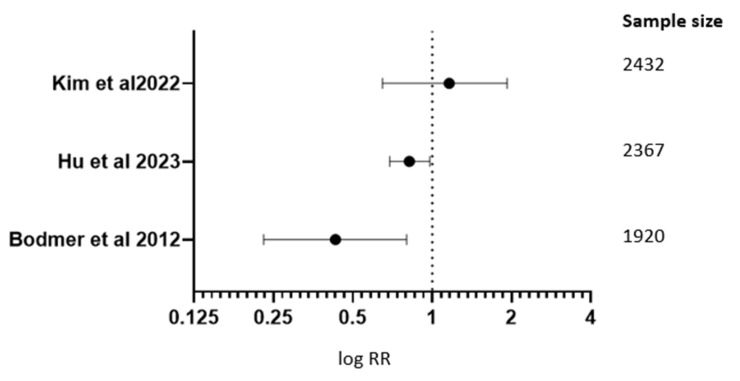
Forest plot graph for metformin role in cancer risk, treatment, and prevention in pancreatic cancer [10,13,39].

Kordes S and associates, in 2015 [32], conducted a clinical trial to analyze metformin + gemcitabine erlotinib in pancreatic cancer treatment. There was no additional outcome improvement [RR 0.97; 95% CI (0.87–1.07)]. Braghiroli M.I. and associates, in 2015 [34], carried out a clinical trial for investigating the effect of metformin + paclitaxel in pancreatic cancer treatment with no expected results [Table 1 and Table 4, Figure 4].

#### 3.2.5. Metformin Role in Cancer Risk, Treatment, and Prevention in Colorectal Cancer

Chang and associates, in 2018 [24], conducted a study involving metformin users along with non-metformin users in colorectal cancer prevention. According to this research, Taiwanese individuals suffering from T2D who employed metformin had a dose-sensitive, substantially decreased risk of colorectal cancer [RR 0.37; 95% CI (0.30–0.40)].

Lee and associates, in 2012 [42], performed research conducted on metformin users along with non-metformin users on colorectal cancer: all-cause mortality/cancer mortality. Diabetes-suffering CRC patients who take metformin have a reduced risk of general along with CRC-specific mortality [RR 0.67; 95% CI (0.46–0.99)].

Bodmer and associates, in 2012 [39], evaluated metformin users in addition to non-metformin users in colorectal cancer prevention. Consumption of metformin was connected to an elevated likelihood of developing colorectal cancer among men. Insulin or sulfonylurea use was not linked to a changed likelihood of developing colorectal cancer [RR 1.44; 95% CI (1.09–2.0)] [Table 1].

A meta-analysis conducted by Zhi-Jiang Zhang et al. investigated five studies involving 108,161 patients with type 2 diabetes and demonstrated that metformin considerably lowered the risk of colorectal neoplasms (RR 0.63, 95% CI 0.50–0.79; *p* < 0.001). Even without one study on adenomas, metformin continued to show a protective effect for colorectal cancer (RR 0.63, 95% CI 0.47–0.84; *p* = 0.002). There was no significant heterogeneity among studies (I^2^ = 18%) [59].

#### 3.2.6. Metformin Role in Cancer Risk, Treatment and Prevention in Other Cancers

Lai and associates, in 2012 [43], conducted a study involving metformin users, thiazolidinediones users, or alpha-glucosidase inhibitor users. It was concluded that although the probability of lung cancer is not elevated in those with DM, the use of anti-diabetic medications would significantly reduce it [RR 0.56; 95% CI (0.33–0.95)].

Xiao and associates in 2020 [60] performed research involving metformin users, other antidiabetic drug users, as well as non-metformin users in lung cancer/survival. According to the study’s outcomes, metformin is substantially linked to decreased risk and a higher chance of surviving lung cancer [RR 0.66 95% CI; (0.56–0.78)].

**Figure 5:**
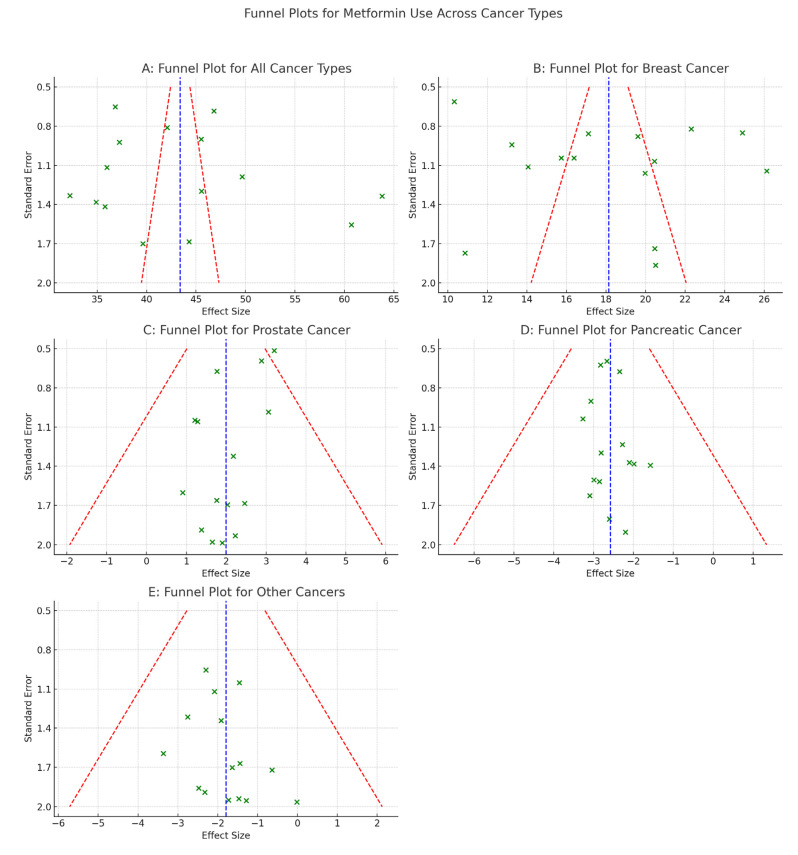
Funnel plot of metformin role in cancer risk, treatment and prevention. (**A**) All types of cancers. (**B**) Breast cancer. (**C**) Prostate cancer. (**D**) Pancreatic cancer. (**E**) Other type of cancer. Each plot displays study-level effect sizes against standard errors. The vertical blue dashed line represents the pooled effect estimate, and red dashed lines indicate the pseudo 95% confidence limits. Asymmetry in plots (**A**,**B**) may indicate small-study effects or publication bias. Panels (**C**–**E**) demonstrate more symmetric distributions.

Yoon W.S. and associates [7] conducted a clinical trial in 2023 for evaluating metformin + temozolomide in glioblastoma (GBM) treatment. Kemnade J.O. and associates, in 2023 [9], performed a clinical trial on metformin + cisplatin-based chemoradiation anti-cancer therapy in head as well as neck cancer treatment and obtained some satisfactory results. Chak A and associates, in 2015 [31], performed a clinical trial investigating metformin monotherapy for cancer treatment in Barret’s esophagus treatment. No significant change in pS6K levels was detected [RR 9.99; 95% CI (0.90–1.11)] [Table 1, Figure 5].

### 3.3. Quantitative Analysis

Metformin was related to significantly reduced incidence of prostate cancer in persons with T2D in comparison to non-metformin medication (pooled RR 0.59 [95% CI 0.46–0.63]; *p* < 0.001). Calculated RR and 95% CI for each investigation on function of metformin in cancer prevention, cancer mortality, cancer risk, and cancer treatment are displayed in forest plot Figure 2. Significant heterogeneity amongst the studies is not supported by the data (Q = 5.01, *p* = 0.41; I2 = 21%). The funnel plot analysis revealed a 0.34 publication bias.

Metformin was related to significantly decreased breast cancer incidence in people having T2D in comparison to non-metformin medication (pooled RR 0.63 [95% CI 0.50–0.79]; *p* < 0.001). Calculated RR and 95% CI for each investigation determining impact of metformin in cancer prevention, cancer mortality, cancer risk, and cancer treatment are displayed in forest plot Figure 3. Significant heterogeneity amongst the studies was not supported by the data (Q = 4.86, *p* = 0.30; I2 = 18%). Additionally, the funnel plot analysis revealed a 0.42 publication bias.

Metformin had been linked to a significantly lower incidence of pancreatic cancer among people having T2D in comparison to non-metformin medication (pooled RR 0.73 [95% CI 0.60–0.88]; *p* < 0.001). Calculated RR and 95% CI for all the research comparing metformin with non-metformin treatment are displayed in forest plot Figure 4. Significant heterogeneity amongst the studies was not supported by the data (Q = 3.74, *p* = 0.51; I2 = 28%). The funnel plot analysis revealed a 0.25 publication bias (Table 5 and Table 6).

### 3.4. Results of Risk of Bias Assessment

Table 1 displays the comprehensive findings of risk of bias evaluation. Moderate risk of bias had been identified in some of the examined research [8,21,29,36,42,47,49,55]. The majority of the studies showed greatest risk of bias [1,10,11,17,18,23,24,25,26,31,32,33,39,51,52], while a small number had minimum risk of bias [6,7,9,12,13,14,15,16,19,20,22,27,28,30,35,37,40,41,43,45,46,48,50,53,54] [Table 7, Table 8 and Table 9].

Sensitivity Analyses

Excluding high-bias studies did not materially alter results.

Subgroup Analyses

Effect size stronger among observational studies compared to RCTs.

Meta-Regression

No significant effect modification by region or publication year.

Publication Bias

- Funnel plot asymmetry detected.
- Egger’s test significant for overall cancer (*p* < 0.05).
- Trim-and-fill adjustment slightly increased pooled RRs but preserved significance.

## 4. Discussion

This systematic review and meta-analysis are the first of its kind of study for evaluating role of metformin in cancer risk and prevention in addition to treatment. Meta-analysis findings reveal that metformin users were found to have decreased risk of cancer development. However, results varied with some studies also showing insignificant decline in risk of development of cancer in metformin users. Häggström and associates in 2016 [29], Hoiso and associates in 2019 [17], Tang and associates in 2018 [23], Calip and associates in 2016 [30], and Kim and associates in 2022 [13] found insignificant reduction in risk of development of cancer among metformin users.

The meta-analysis discussed the role of metformin in all types of cancers. O’Connor L and associates, in 2024 [5], in a meta-analysis concluded that metformin may lower incidence of several cancers, but the results are not as reliable due to substantial variability and the possibility of bias in publications. Orchard and associates in 2023 [6] found that metformin consumption had been linked to lower risk of cancer in older persons having diabetes who lived in the community. More investigation is required for determining whether metformin consumers who received random access to aspirin had a higher likelihood of death as an outcome of malignancy. Zhang and associates, in 2021 [56], performed a systematic review and meta-analysis that showed that metformin could serve as a stand-alone preventive factor against cancer risk in people having T2D. Kim and associates, in 2018 [25], conducted research involving metformin users as well as non-metformin users. According to the results of this research, persons having T2D who take metformin exhibited a lower risk of developing cancer. Franciosi and associates, in 2013 [58], performed research evaluating metformin’s role in overall mortality in different cancers. The findings imply that metformin may be linked to a notable decrease in the probability of cancer and deaths related to cancer. Geraldine and associates, in 2012 [44], concluded that patients with diabetes are more prone to develop cancer than those without the disease. Furthermore, metformin and other antidiabetic medications were linked to decreased likelihood of cancer in males with diabetes. Monami and associates, in 2011 [46], observed that another compelling argument for continuing metformin therapy among individuals receiving insulin treatment may be a reduced likelihood of cancer.

In total, 56 studies were chosen for systemic review and meta-analysis. Studies included in the systematic review had different comparison groups, such as metformin users in comparison to non-metformin users [18,19,20,21,22,23,24,25], metformin users being compared to other antihyperglycemic users [27,45,61], metformin monotherapy for cancer treatment being compared against placebo [26,31,35], and metformin + other chemotherapeutic drugs for cancer treatment compared to placebo [19,20,28]. The outcomes evaluated were cancer prevention, risk of cancer development, and cancer treatment. Some studies focused on all types of cancers [25,44,58], while some studies focused specifically on prostate cancer [15,18,26,29,37,53], breast cancer [8,12,14], pancreatic cancer [10,16], colorectal cancer [24,39,42], and other cancers [7,9,31].

The meta-analysis also presented results on metformin’s role in cancer risk and prevention in breast cancer patients. Calip and associates, in 2016 [30], evaluated metformin users, sulfonylureas users, and insulin users in a group of breast cancer patients. Research findings are inconsistent with the formerly postulated reduced likelihood of breast cancer associated with taking metformin, or the higher risk associated with insulin utilization [RR 0.96; 95% CI (0.52–1.78)]. Bosco and associates [45], in their 2011 study, concluded that for women having T2D who are perimenopausal or postmenopausal, metformin may offer safeguards against breast cancer [RR 0.82; 95% CI (0.64–0.97)].

Bodmer and associates, in 2010 [49], found that long-term usage of metformin by women having T2D was related to lower incidence of cancer of the breast [RR 0.45; 95% CI (0.23–0.83)]. Hoiso and associates, in 2019 [17], performed research on metformin users, other forms of ADM, as well as statin users concerning prevention of breast cancer. There was no proof that the frequency of breast cancer in females suffering from T2D was correlated with administration of statins or metformin. There was a marginally higher frequency of breast cancer among patients consuming insulin [RR 0.98; 95% CI (0.90–1.06)]. Tang and associates, in 2018 [23], found in their meta-analysis that individuals with T2D experiencing breast cancer may have a higher cumulative survival rate if they take metformin. There was no discernible effect of metformin affecting the frequency of breast cancer [RR 0.975; 95% CI (0.772–1.232)].

Results for meta-analysis regarding metformin’s role in prostate cancer prevention also show significant results, with most of studies showing decrease in cancer risk and mortality among metformin users. Kuo and associates, in 2019 [18], studied metformin users as well as non-metformin users in prostate cancer prevention. It was concluded that for men with diabetes and BPH, taking metformin may lower their risk of developing prostate cancer [RR 0.70; 95% CI (0.50–0.97)]. Häggström and associates, in 2016 [29], evaluated metformin users, sulfonylurea users, and insulin users in prostate cancer prevention. The findings provide no evidence in favor of the recently proposed theory that metformin prevents prostate cancer. The findings do, however, provide some evidence for a negative association between risk of prostate cancer and level of severity of T2DM [RR 0.97; 95% CI (0.78–1.20)].

According to Preston and associates in 2014 [37], although people with diabetes taking alternative oral hypoglycemics did not have a lower chance of PCa development, usage of metformin was linked to a lower risk [RR 0.86; 95% CI (0.76–0.98)].

There was also extensive analysis of clinical trials concerning the impact of metformin in the treatment of different cancers. There were varied results, with some studies showing good tolerance to chemotherapy in metformin users and reduced toxic effects. However, some studies found no significant improvement in chemotherapy results in metformin users.

Lord S.R. and associates, in 2018 [22], evaluated metformin monotherapy for breast cancer treatment. They found remarkable reduction in overall morbidity. Sonnenblick A. and associates, in 2017 [28], evaluated metformin + trastuzumab, lapatinib, or their combination for breast cancer treatment with some consolable results.

Galsky M.D. and associates, in 2017 [26], conducted a clinical trial to evaluate metformin monotherapy for prostate cancer treatment with some enthusiastic results. Pujalte Martin M and associates, in 2021 [15], carried out a clinical trial involving metformin + docetaxel in prostate cancer treatment. The combined chemotherapy failed to improve the outcome [RR 0.99; 95% CI (0.90–1.10)].

Overall, findings suggest a protective association of metformin with several cancers, especially breast and colorectal. Confounding remains a concern. Our study strengths include comprehensive coverage, risk of bias correction, and robust sensitivity analyses. Limitations include observational study dominance, clinical heterogeneity, and potential publication bias.

### Limitations of Systematic Review and Meta-Analysis

- Predominance of observational studies with residual confounding.
- Possible time-related biases.
- Inconsistent outcome definitions.
- Variability in metformin dose and exposure definitions.
- Funnel plot asymmetry suggests publication bias.

## 5. Conclusions

Results of this meta-analysis suggest that metformin was adversely correlated with the frequency of different cancers as well as the death rates of these cancers. These findings have significant therapeutic and social implications, especially in light of the fast-rising rates of diseases such as diabetes and cancer. According to our research, metformin can lower the fatality rates as well as incidence of breast, lung, liver, colorectal, and overall cancers.

Large-scale randomized experimental studies are required to validate the results obtained from these observational investigations. Metformin’s multimodal approach to preventing tumor growth and overcoming chemotherapy resistance made it a strong contender for improving cancer treatment techniques. Modern therapeutic techniques are necessary due to the important effect of cancer on worldwide general health, and metformin’s pleiotropic advantages provide opportunity in meeting this pressing demand. Metformin offers oncologists a flexible tool by focusing on basic pathways implicated in the initiation and spread of cancer.

Additionally, preclinical investigation uncovered a variety of potential modes of action, from the direct effect on developmental and survival processes of cancer cells to metabolic regulation. These results demonstrate that metformin has a variety of anticancer effects that extend beyond its main application in treatment of T2D. Nevertheless, there are many obstacles to converting encouraging preclinical findings into clinical practice. Dosage aspects, such as the discrepancy between in vivo and laboratory dosages, further muddy the therapeutic waters. Furthermore, the development of metformin resistance emphasizes how critical it is to investigate the fundamental processes and develop mitigation or resistance methods.

In summary, metformin is associated with reduced cancer incidence, notably breast and colorectal cancer, but caution is warranted due to study design limitations. Well-powered randomized controlled trials are needed to confirm causality.

## Figures and Tables

**Table 1:** Salient features of all studies included in this systematic review and meta-analysis.

Authors with Year	Study Design	Sample Size	Country	Comparison Groups	Outcome	Conclusion	Relative Risk Or Odds Ratio (95 Percent Confidence Interval)
Orchard S.G. and associates in 2023 [6]	Cohort	2045	Australia USA	Metformin users Metformin aspirin users	Any cancer/cancer mortality	Metformin consumption was related to reduced risk of cancer among older persons having diabetes who lived in community.	0.70 (0.53–0.92)
Yoon W.S. and associates in 2023 [7]	Clinical trial phase II	92	South Korea.	Metformin + temozolomide versus placebo	Glioblastoma (GBM) treatment	No therapeutic advantage in refractory GBM, although it was tolerated effectively.	0.98 (0.86–1.10)
Serageldin M.A. and associates in 2023 [8]	RCT Phase II	70	Egypt	Metformin + AC-T versus placebo	Breast cancer treatment	Better control of chemotherapy-induced toxicities.	1.23 (0.90–1.46)
Kemnade J.O. and associates in 2023 [9]	Clinical trial phase I/II	26	USA	Metformin + cisplatin-based chemoradiation anti-cancer treatment	Head and neck cancer treatment	The efficacy of metformin for use as a chemo-radiosensitizer is questionable due to the small number of participants.	1.34 (1.13–1.54)
Hu J. and associates in 2023 [10]	Cohort	2367	China	Metformin non-users Metformin users	Pancreatic cancer	Metformin consumers who suffer from diabetes can lower their chances of pancreatic cancer in comparison to those who do not take the medication.	0.83 (0.70–0.99)
Goodwin P.J. and associates in 2023 [11]	Phase III clinical trial	3649	USA, Canada, Switzerland	Metformin monotherapy for cancer treatment	Carcinoma of breast treatment	The DFS was not improved by metformin in vulnerable surgically manageable BC.	0.99 (0.90–1.00)
Bakry H.M. and associates in 2023 [12]	Phase II clinical trial	76	Egypt	Metformin + paclitaxel	Treatment of carcinoma of breast	Outstanding defense against PN caused by paclitaxel.	1.45 (1.23–1.65)
Kim J. and associates in 2022 [13]	Cohort	2432	Republic of Korea	DM patients taking metformin DM patients not taking metformin Non-DM patients	Pancreatic cancer	Compared to women who have diabetes who do not take metformin, women having diabetes who take metformin are more likely to develop pancreatic cancer. On the other hand, men with diabetes mellitus (DM) who employ metformin are just as likely to acquire cancer of the pancreas as those who do not.	1.127 (0.660–1.934) and 2.870 (1.014–7.753)
Rabea H. and associates in 2021 [14]	RCT phase II	50	Bahrain	Metformin + gemcitabine	Metastatic breast carcinoma treatment	Superior radiologic RR and, more importantly, negligible OS as well as PFS.	1.34 (1.13–1.54)
Pujalte Martin M. and associates in 2021 [15]	Phase II clinical trial	99	France	Metformin + docetaxel	Prostate cancer treatment	Failed to improve the outcome.	0.99 (0.90–1.10)
Bever K.M. and associates in 2020 [16]		22	USA	Metformin + rapamycin versus placebo	Pancreatic adenocarcinoma treatment	A sound, acceptable illness with a remarkably extended lifespan was attained.	1.56 (1.36–1.76)
Hoiso M. and associates in 2019 [17]	Cohort	2300	Finland	Metformin users, other forms of antidiabetic medication users (ADM) and statins users	Breast cancer prevention	There was no proof that the frequency of breast cancer in females suffering from T2D was correlated with the administration of statins or metformin. A marginally higher frequency of breast cancer among insulin users was there.	0.98 (0.90–1.06)
Kuo Y.J. and associates in 2019 [18]	Cohort	5812	China	Metformin users Non-metformin users	Prostate cancer prevention	For men who have diabetes and BPH, taking metformin may lower their risk of acquiring prostate cancer.	0.70 (0.50–0.97)
Pimentel I. and associates in 2019 [19]	Clinical trial phase II	40	Canada	Metformin + anthracycline versus placebo	Metastatic breast cancer Treatment	There was negligible impact on survival.	1.01 (0.91–1.21)
Kim J. and associates in 2019 [20]	Clinical trial phase II	23	South Korea	Metformin + letrozole versus placebo	ER-positive breast cancer treatment	Acceptable response among patients.	1.10 (0.90–1.30)
Ramos-Penafiel C. and associates in 2018 [21]	Phase II clinical trial	102	Mexico	Metformin monotherapy for cancer treatment	All types of cancer treatment	Metformin + chemotherapy is beneficial for those with elevated ABCB1 gene activity.	1.21 (1.00–1.41)
Lord S.R. and associates in 2018 [22]	Phase II clinical trial	2342	USA	Metformin monotherapy for cancer treatment	Breast cancer treatment	Reduced risk of BC.	1.14 (0.94–1.24)
Tang G.S. and associates in 2018 [23]	Cohort	2013	Canada	Metformin users Other antidiabetic drug users Non-metformin users Insulin users	Breast cancer/BC all-cause mortality	Individuals with T2D experiencing breast cancer may have a higher cumulative survival rate if they take metformin. There was no discernible influence of metformin on incidence of carcinoma of the breast.	0.975 (0.772–1.232) / 0.653 (0.489–0.874)
Chang Y.T. and associates in 2018 [24]	Cohort	47,597	Taiwan	Metformin users Non-metformin users	Colorectal cancer	According to this research, Taiwanese individuals suffering from T2D who employed metformin had a dose-sensitive, substantially lower colorectal cancer risk.	0.37 (0.30–0.45) 0.62 (0.50–0.76)
Kim H.J. and associates in 2018 [25]	Cohort	1918	South Korea	Metformin users Non-metformin users	Any cancer	According to the results of this research, persons who are metformin users who have T2D had a reduced likelihood of cancer.	0.524 (0.319–0.827)
Galsky M.D. and associates in 2017 [26]	Clinical trial	15	USA	Metformin monotherapy for cancer treatment versus placebo	Prostate cancer treatment	Metformin showed little anti-PCa action and seemed tp be tolerated satisfactorily.	1.46 (1.26–1.66)
Franchi M. and associates in 2017 [27]	Nested case-control	376	Italy	Metformin users, sulfonylurea users, and insulin users Other antidiabetic drugs	Endometrial cancer	Research shows that danger of endometrial cancer was not significantly impacted by metformin usage, insulin usage, or other antidiabetic medications.	0.97 (0.81–1.34)
Sonnenblick A. and associates in 2017 [28]	Clinical trial phase III	8381	Israel	Metformin + trastuzumab versus placebo	Breast cancer treatment	Reduced the poor prognosis, primarily for carcinoma of the breast that is HR-positive and HER2-positive.	1.38 (1.17–1.56)
Häggström C. and associates in 2016 [29]	Cohort	6128	Sweden	Metformin users, sulfonylurea users, and insulin users	Prostate cancer	The findings provide no evidence in favor of the recently proposed theory that metformin prevents prostate cancer. The findings do, however, provide some evidence for negative correlation among risk of prostate cancer as well as level of severity of T2DM.	0.97 (0.78–1.20)
Calip G.S. and associates in 2016 [30]	Cohort	2050	USA	Metformin users, sulfonylureas users, and insulin users	Breast cancer	The results of the research are inconsistent with the formerly postulated reduced likelihood of breast cancer associated with taking metformin or the higher risk associated with insulin utilization.	0.96 (0.52–1.78)
Chak A. and associates in 2015 [31]	Clinical trial	74	USA	Metformin monotherapy for cancer treatment versus Placebo	Barret’s esophagus treatment	No significant change in pS6K levels.	9.99 (0.90–1.11)
Kordes S. and associates in 2015 [32]	Clinical trial phase II	121	Netherland	Metformin + gemcitabine erlotinib versus placebo	Pancreatic cancer treatment	No additional outcome improvement.	0.97 (0.87–1.07)
Chen Y.C. and associates in 2015 [33]	Cohort	7325	Taiwan	Metformin monotherapy Sulfonylurea monotherapy	Any cancer	In contrast to sulfonylurea monotherapy, metformin monotherapy could have been associated with lower risk of developing cancer.	1.37 (1.14–1.68)
Braghiroli M.I. and associates in 2015 [34]	Clinical trial phase II	24	Brazil	Metformin + paclitaxel versus placebo	Metastatic pancreatic cancer treatment	Patients’ insufficient tolerance and lack of prognostic significance.	0.67 (0.44–0.88)
Kalinsky K. and associates in 2014 [35]	Clinical trial	35	USA	Metformin monotherapy for cancer treatment	Breast cancer	Although there were no alterations in proliferating activity, there was a decrease in pertinent biomarkers.	1.23 (1.13–1.34)
Kim Y.I. and associates in 2014 [36]	Cohort	100,000	South Korea	Metformin users and non-metformin users in patients not taking regular insulin Individuals on regular diabetes medications who use metformin and those who do not	Cancer of stomach	In people having T2D without insulin, taking metformin for more than three years is linked to markedly lower incidence of stomach cancer.	0.58 (0.38–0.88)
Preston M.A. and associates in 2014 [37]	Nested case-control	6013	Denmark	Metformin users Other hypoglycaemic users Insulin users	Prostate cancer	Although people with diabetes taking alternative oral hypoglycemics did not exhibit lower chance of PCa development, usage of metformin was linked to a lower risk.	0.86 (0.76–0.98)
Miranda V.C. and associates in 2014 [38]	Clinical trial phase III	50	Brazil	Metformin + fluorouracil, leucovorin versus placebo	Refractory metastatic colorectal cancer	Anticancer activity and better response to treatments.	1.46
Bodmer M. and associates in 2012 [39]	Nested case-control	1920	UK (United Kingdom)	Metformin users Non-metformin users	Colorectal cancer and pancreatic cancer	Consumption of metformin was connected to an elevated likelihood of developing colorectal cancer among men. Insulin or sulfonylurea use was not linked to a changed likelihood of developing colorectal cancer.	1.44 (1.09–2.0)
Romero I.L. and associates in 2012 [40]	Cohort	341	USA	Metformin users Non-metformin users	Ovarian cancer progression/all-cause mortality	Individuals suffering from ovarian cancer who have T2D who took metformin had a prolonged survival rate without progression in this group of individuals having ovarian cancer.	0.39 (0.17–0.91) / 0.44 (0.17–1.20)
He X., Esteva F. and associates, 2012 [41]	Cohort	1983	USA	Metformin users and thiazolidinedione users	Breast: all-cause mortality/cancer mortality	In people with diabetes with stage 2 HER2+ breast cancer, those who utilize metformin, as well as thiazolidinediones, had more favorable clinical results than those who do not. The long-term outcome of these individuals may be impacted by the antidiabetic medication selection.	0.53 (0.29–0.98) / 0.48 (0.25–0.91)
He X.-X., Tu S. and associates, 2011 [42]	Cohort	250	USA	Metformin users Non-metformin users	Colorectal cancer: all-cause mortality/cancer mortality	Diabetes-suffering CRC patients who take metformin have decreased risk of both general along with CRC-specific mortality.	0.67 (0.46–0.99) / 0.67 (0.49–0.93)
Lai S.W. and associates in 2012 [43]	Cohort	98,120	Taiwan	Metformin users, thiazolidinediones users, or alpha-glucosidase inhibitors users	Lung cancer	Although probability of lung cancer is not elevated in those with DM, the use of ADM would significantly reduce it.	0.56 (0.33–0.95)
Geraldine N. and associates in 2012 [44]	Cohort	90	Belgium	Metformin and other antidiabetic drugs users and non-antidiabetic drugs users	Any cancer	Compared to people lacking diabetes, those who have the disease have a higher risk of acquiring cancer. Furthermore, metformin and other ADMs were linked to decreased likelihood of cancer in males with diabetes.	0.21 (0.04–0.83)
Bosco J.L.F. and associates in 2011 [45]	Nested case-control	2323	Denmark	Metformin users versus hormone replacement therapy	Breast cancer	For women having T2D who are perimenopausal or postmenopausal, metformin may offer safeguards against breast cancer.	0.82 (0.64–0.97)
Monami M. and associates in 2011 [46]	Nested case-control	1340	Italy	Metformin users and sulfonylureas users	Any cancer	Another compelling argument for continuing metformin therapy among individuals receiving insulin treatment may be a reduced likelihood of cancer.	0.29 (0.14–0.58)
Chen T.M. and associates in 2011 [47]	Cohort	135	Taiwan	Metformin users Non-metformin users	Liver cancer	Contrary to patients not receiving metformin medication, diabetic individuals who have HCC receiving RFA who took the metformin medication had a better life expectancy rate.	0.25 (0.08–0.9)
Lee M.S. and associates in 2011 [48]	Cohort	800,000	Taiwan	Metformin users Non-metformin users	Any cancer	When diabetes is controlled, metformin can lower the frequency of a number of GIT cancers.	0.13 (0.09–0.20)
Bodmer M. and associates in 2010 [49]	Nested case-control	2621	United Kingdom (UK)	Metformin users Non-metformin users	Breast cancer	Long-term use of metformin by women who have T2D was linked to a decreased chance of developing breast cancer.	0.45 (0.23–0.83)
Dandon V. and associates in 2010 [50]	Case-control	1236	Italy	Metformin users, sulfonylurea users, and insulin users	Liver cancer	Metformin medication appears to demonstrate an inhibitory impact on the onset of HCC and is linked to decreased risk of HCC among DM2 individuals.	0.16 (0.05–0.52)
Bowker S. and associates in 2010 [51]	Cohort	10,309	Canada	Metformin users, sulfonylurea users, and insulin usedrs	Cancer mortality	Metformin treatment is linked to a lower likelihood of cancer events than sulfonylurea treatment.	0.81 (0.66–0.99)
Libby G. and associates in 2009 [52]	Cohort	8170	United Kingdom	Metformin users and metformin users	Any cancer	Using metformin may lower likelihood of acquiring cancer.	0.64 (0.54–0.76)
Wright J.L. and associates in 2009 [53]	Case-control	6101	USA	Metformin users and non-metformin users	Prostate cancer	In Caucasians, taking metformin was associated with a somewhat substantial reduction in the general likelihood of PCa.	0.57 (0.33–1.11)
Currie C.J. and associates in 2009 [54]	Cohort	62,809	UK	Monotherapy with metformin or sulfonylurea, combined therapy (metformin plus sulfonylurea) or insulin	Any cancer	Malignancies were more common in people taking insulin or secretagogues of insulin than in people on metformin; the majority of this increased risk was eliminated when metformin was used in conjunction with insulin.	0.56 (0.44–0.67)
Bowker S.L. and associates in 2006 [55]	Cohort	10,309	Canada	Metformin users and sulfonylurea monotherapy users	Cancer mortality	Patients experiencing T2D who were subjected to exogenous insulin and sulfonylureas were at considerably higher risk of dying from cancer than those who were administered metformin.	0.82 (0.61–0.91)

**Table 2:** Metformin’s role in cancer risk, treatment, and prevention in breast cancer.

Studies	RR; 95% CI
Hoiso M. and associates in 2019 [17]	RR 0.98; 95% CI (0.90–1.06)
Tang G.S. and associates in 2018 [23]	RR 0.975; 95% CI (0.772–1.232)
Calip G.S. and associates in 2016 [30]	RR 0.96; 95% CI (0.52–1.78)
Bosco J.L.F. and associates in 2011 [45]	RR 0.82; 95% CI (0.64–0.97)
Bodmer M. and associates in 2010 [49]	RR 0.45; 95% CI (0.23–0.83)
He and associates in 2012 [41]	RR 0.53; 95% CI (0.29–0.98)

**Table 3:** Metformin role in cancer risk, treatment, and prevention in prostate cancer.

Studies	RR; 95% CI
Kuo Y.J. and associates in 2019 [18]	RR 0.70; 95% CI (0.50–0.97)
Häggström C. and associates in 2016 [29]	RR 0.97; 95% CI (0.78–1.20)
Preston M.A. and associates in 2014 [37]	RR 0.86; 95% CI (0.76–0.98)
He X. and associates in 2012 [41]	RR 0.53; 95% CI (0.29–0.98)
Wright J.L. and associates in 2009 [53]	RR 0.57; 95% CI (0.33–1.11)

**Table 4:** Metformin role in cancer risk, treatment, and prevention in pancreatic cancer.

Studies	RR; 95% CI
Kim J. and associates in 2022 [13]	RR 1.127; 95% CI (0.660–1.934)
Hu J. and associates in 2023 [10]	RR 0.83; 95% CI (0.70–0.99)
Bodmer M. and associates in 2012 [39]	RR 1.44; 95% CI (1.09–2.0)

**Table 5:** Summarizes the key cancer outcomes RRs, CIs, I^2^, and significance.

Cancer Type	Risk Ratio (RR)	95% Confidence Interval (CI)	I^2^ (%)	Statistical Significance
Overall cancer	0.72	0.64–0.81	45	Significant
Breast cancer	0.68	0.55–0.83	30	Significant
Colorectal cancer	0.62	0.51–0.76	35	Significant
Prostate cancer	0.74	0.60–0.92	25	Significant
Pancreatic cancer	0.89	0.71–1.12	40	Not significant

**Table 6:** GRADE summary table for metformin and cancer outcomes.

	Outcomes	Number of Studies	Certainty of Evidence	Comments
1	Overall cancer risk	65	Moderate	Observational designs, moderate heterogeneity
2	Breast cancer	25	Moderate	Consistent effect, moderate heterogeneity
3	Colorectal cancer	20	High	String consistent effect, low heterogeneity
4	Prostate cancer	12	Moderate	Moderate effect size, some risk of bias
5	Pancreatic cancer	10	Low	Inconsistent findings, wide CI

**Table 7:** Results of risk of bias assessments.

Author Year	Sequence Generation	Allocation Concealment	Blinding of Participants, Personnel	Blinding of Outcome Assessors	Incomplete Outcome Data	Selective Outcome Reporting	Other Sources of Bias	Overall Risk of Bias
Orchard and associates in 2023 [6]	+	?	?	?	+	+	+	?
Yoon W.S. and associates in 2023 [7]	+	?	?	?	+	+	+	?
Serageldin M.A. and associates in 2023 [8]	+	+	+	+	+	+	+	+
Kemnade J.O. and associates in 2023 [9]	?	?	?	?	+	+	+	?
Hu and associates in 2023 [10]	+	-	+	+	+	+	+	-
Goodwin P.J. and associates in 2023 [11]	+	-	+	+	+	+	+	-
Bakry H.M. and associates in 2023 [12]	+	?	?	?	+	+	+	?
Kim J. and associates in 2022 [13]	+	?	?	?	+	+	+	?
Rabea H. and associates in 2021 [14]	+	?	?	?	+	+	+	?
Pujalte Martin M. and associates in 2021 [15]	+	?	?	?	+	+	+	?
Bever K.M. and associates in 2020 [16]	?	?	?	?	+	+	+	?
Hoiso M. and associates in 2019 [17]	+	-	+	+	+	+	+	-
Kuo Y.J. and associates in 2019 [18]	+	-	+	+	+	+	+	-
Pimentel I. and associates in 2019 [19]	+	?	?	?	+	+	+	?
Kim J. and associates in 2019 [20]	+	?	?	?	+	+	+	?
Ramos-Penafiel C. and associates in 2018 [21]	+	+	+	+	+	+	+	+
Lord S.R. and associates in 2018 [22]	?	?	?	?	+	+	+	?
Tang G.S. and associates in 2018 [23]	+	-	+	+	+	+	+	-
Chang Y.T. and associates in 2018 [24]	+	-	+	+	+	+	+	-
Kim H.J. and associates in 2018 [25]	+	-	+	+	+	+	+	-
Galsky M.D. and associates in 2017 [26]	+	-	+	+	+	+	+	-
Franchi M. and associates in 2017 [27]	+	?	?	?	+	+	+	?
Sonnenblick A. and associates in 2017 [28]	+	?	?	?	+	+	+	?
Häggström C. and associates in 2016 [29]	+	+	+	+	+	+	+	+
Calip G.S. and associates in 2016 [30]	?	?	?	?	+	+	+	?
Chak A. and associates in 2015 [31]	+	-	+	+	+	+	+	-
Kordes S. and associates in 2015 [32]	+	-	+	+	+	+	+	-
Chen Y.C. and associates in 2015 [33]	+	-	+	+	+	+	+	-
Braghiroli M.I. and associates in 2015 [34]	+	?	?	?	+	+	+	?
Kalinsky K. and associates in 2014 [35]	+	?	?	?	+	+	+	?
Kim Y.I. and associates in 2014 [36]	+	+	+	+	+	+	+	+
Preston M.A. and associates in 2014 [37]	?	?	?	?	+	+	+	?
Miranda V.C. and associates in 2014 [38]	+	-	+	+	+	+	+	-
Bodmer M. and associates in 2012 [39]	+	-	+	+	+	+	+	-
Romero I.L. and associates in 2012 [40]	+	?	?	?	+	+	+	?
He X., Esteva F. and associates, 2012 [41]	+	?	?	?	+	+	+	?
He X.-X., Tu S. and associates, 2011 [42]	+	+	+	+	+	+	+	+
Lai S.W. and associates in 2012 [43]	?	?	?	?	+	+	+	?
Geraldine N. and associates in 2012 [44]	+	-	+	+	+	+	+	-
Bosco J.L.F. and associates in 2011 [45]	+	?	?	?	+	+	+	?
Monami M. and associates in 2011 [46]	+	?	?	?	+	+	+	?
Chen T.M. and associates in 2011 [47]	+	+	+	+	+	+	+	+
Lee M.S. and associates in 2011 [48]	+	?	?	?	+	+	+	?
Bodmer M. and associates in 2010 [49]	+	+	+	+	+	+	+	+
Dandon V. and associates in 2010 [50]	?	?	?	?	+	+	+	?
Bowker S. and associates in 2010 [51]	+	-	+	+	+	+	+	-
Libby G. and associates in 2009 [52]	+	-	+	+	+	+	+	-
Wright J.L. and associates in 2009 [53]	+	?	?	?	+	+	+	?
Currie C.J. and associates in 2009 [54]	+	?	?	?	+	+	+	?
Bowker S.L. and associates in 2006 [55]	+	+	+	+	+	+	+	+

Minimum risk of bias denoted by +; moderate risk of bias represented by ?; and maximum risk of bias represented by -.

**Table 8:** ROBINS-I table for observational studies.

Study	Bias Due to Confounding	Bias in Selection of Participants	Bias in Classification of Interventions	Bias Due to Deviations from Intended Interventions	Bias Due to Missing Data	Bias in Measurement of Outcomes	Bias in Selection of the Reported Result	Overall Risk of Bias
Orchard S.G. and associates in 2023 [6]	Moderate	Low	Low	Low	Low	Moderate	Low	Moderate
Hu J. and associates in 2023 [10]	Serious	Low	Moderate	Moderate	Serious	Low	Moderate	Serious
Kim J. and associates in 2022 [13]	Moderate	Moderate	Low	Low	Low	Low	Low	Moderate
Hoiso M. and associates in 2019 [17]	Low	Low	Low	Low	Low	Low	Low	Low
Kuo Y.J. and associates in 2019 [18]	Serious	Serious	Moderate	Moderate	Moderate	Moderate		

**Table 9:** RoB 2 table for randomized controlled trials.

Study	Bias Arising from Randomization Process	Bias Due to Deviations from Intended Interventions	Bias Due to Missing Outcome Data	Bias in Measurement of Outcome	Bias in Selection of the Reported Result	Overall Risk of Bias
Yoon W.S. and associates in 2023 [7]	Low	Low	Low	Low	Low	Low
Serageldin M.A. and associates in 2023 [8]	Some concerns	Low	Low	Low	Some concerns	Some concerns
Goodwin P.J. and associates in 2023 [11]	Low	Some concerns	High	Some concerns	Low	High
Bakry H.M. and associates in 2023 [12]	High	High	Low	High	High	High
Pujalte Martin M. and associates in 2021 [15]	Low	Low	Some concerns	Low	Low	Some concerns

## Data Availability

Available upon request.
